# 4333 The role of the L-type calcium channel, Cav1.3, in motor and associative learning

**DOI:** 10.1017/cts.2020.99

**Published:** 2020-07-29

**Authors:** Aislinn Joanmarie Williams, Marisol Lauffer, Hsiang Wen, Bryn Myers

**Affiliations:** 1 University of Iowa Institute for Clinical and Translational Science

## Abstract

OBJECTIVES/GOALS: Genetic variation in L-type voltage-gated calcium channels, including Ca_V_1.3, is associated with increased risk for psychiatric disorders including bipolar disorder and schizophrenia. Additionally, rare mutations in Ca_V_1.3 have been linked to epilepsy, developmental delay, and autism. Deletion of Ca_V_1.3 in mice is associated with impaired consolidation of contextual fear conditioning. Some studies have also observed affective behavior deficits in Ca_V_1.3-deficient mice, but other studies have not found affective phenotypes, perhaps due to differences in genetic backgrounds, sex ratios, or task protocols. Ca_V_1.3 is important for slow afterhyperpolarization in hippocampal and amygdala neurons, which prevents excessive firing in response to sustained excitatory input, and Ca_V_1.3-deficient amygdala neurons exhibit hyperexcitability and impaired LTP. Ca_V_1.3 is also expressed in the cerebellum, but its functional role there is not well understood. Given its importance in shaping neuronal activity in the hippocampus and amygdala, we hypothesized that loss of Ca_V_1.3 would cause abnormalities in motor learning as well as affective and cognitive behaviors. METHODS/STUDY POPULATION: Wild-type (WT), haploinsufficient (Hap), and knockout (KO) mice were maintained on a congenic C57BL/6NTac genetic background and were subjected to behavioral tasks including open field, rotarod, ErasmusLadder, elevated zero maze, forced swim test, and tail suspension test. Data were analyzed with sexes combined and with sexes separated to assess for sex as a biological variable. Studies were analyzed by one-way ANOVA, two-way ANOVA, or generalized linear mixed model, where appropriate. RESULTS/ANTICIPATED RESULTS: Ca_V_1.3 KO was associated with impaired motor learning in the rotarod task (p < 0.05), as well as impaired associative learning in the ErasmusLadder task (p < 0.01), despite intact locomotor function on both tasks. When examined by sex, the rotarod phenotypes were driven by motor learning impairments in males (both Hap and KO, p < 0.05 and p < 0.01, respectively), whereas the ErasmusLadder associative learning phenotypes were present in both sexes only in the KO condition, consistent with previously reported impairments in Ca_V_1.3-deficient mice in consolidation of contextual fear conditioning. Although KO mice learned the motor aspects of the ErasmusLadder task, they learned more slowly. They also failed to learn start cues, which requires intact associative learning. No differences were observed in overall exploration or locomotor activity in open field or elevated zero maze. Analyses from affective tasks are ongoing. DISCUSSION/SIGNIFICANCE OF IMPACT: These preliminary studies provide new evidence that Ca_V_1.3 is important for the function of neural circuits involved in motor learning, and concur with previous data showing its involvement in associative learning. Our data differ slightly from previous studies of Ca_V_1.3 in motor learning, which could be attributable to differences in task protocols and/or genetic background. These results highlight the importance of Ca_V_1.3 in a variety of behaviors, which may help explain why variation in Ca_V_1.3 expression and function has pleiotropic effects in humans.

